# A Cross-sectional study of stand-alone Percutaneous Coronary Intervention in a Nigerian Cardiac Catheterization Laboratory

**DOI:** 10.1186/1471-2261-14-8

**Published:** 2014-01-16

**Authors:** Adeyemi Johnson, Bode Falase, Ifeoluwa Ajose, Yemi Onabowale

**Affiliations:** 1 First Cardiology Consultants, Ikoyi, Lagos, Nigeria; 2 Cardiothoracic Division, Department of Surgery, Lagos State University College of Medicine, Lagos State University Teaching Hospital, Ikeja, Lagos, Nigeria; 3 Reddington Multispecialty Hospital, Victoria Island, Lagos, Nigeria

## Abstract

**Background:**

There is a paucity of diagnostic and therapeutic facilities in Nigeria to confirm coronary artery disease and offer appropriate interventional therapy. There is now a private cardiac catheterization laboratory in Lagos but as there are no sustained Open Heart Surgery programmes, percutaneous coronary interventions are currently being performed without surgical backup. This study was designed to assess results of stand-alone percutaneous coronary intervention (PCI) as currently practiced in Lagos, Nigeria.

**Methods:**

This cross-sectional study was conducted between July 2009 and July 2012. The study included all patients that underwent PCI in Lagos. Data was extracted from a prospectively maintained database.

**Results:**

Coronary artery disease was confirmed in 80 (52.6%) of 152 Nigerians referred with a diagnosis of Ischaemic Heart Disease. There were 53 males (66.2%) and 27 females (33.8%). The average age was 60.3 +/−9.6 years and average euroscore was 4.5 +/−3.1. Of the 80 patients, 77 (96.3%) had significant stenoses and were candidates for revascularization. Distribution of significant stenoses was one in 32 patients (41.5%), two in 11 patients (14.3%), three in 19 patients (24.7%), four in 13 patients (16.9%) and five in 2 patients (2.6%). PCI was performed in 48 (62.3%) of the patients eligible for revascularization as the coronary anatomy in the remaining patients was not suitable for PCI. The indication for PCI was for myocardial infarction or unstable angina in 39 patients (81.2%). PCI was performed with PTCA plus stenting in 41 patients (85.4%) and with PTCA alone in 7 patients (14.6%) with good angiographic results. Overall 29 of the 48 patients (60.4%) had complete revascularization of significant stenoses. Complications of PCI were bleeding that required blood transfusion in 1 patient (2.1%), minor femoral haematomas in 2 patients (4.2%), and a major adverse clinical event in 1 patient (2.1%).

**Conclusion:**

A stand-alone PCI programme has been developed in Lagos, Nigeria. Both elective and urgent PCIs have been performed with no mortalities and a low complication rate. Increased volumes will however accrue and complete revascularization rates would be improved with the establishment of Open Heart Surgery programmes to provide CABG as back-up for PCI and alternate therapy for more complex lesions.

## Background

Initially thought to be very uncommon in the West African population, it has been noted that there is now a rising incidence of coronary artery disease [1-5]. There has unfortunately been a paucity of diagnostic and interventional facilities in most West African countries, including Nigeria. Efforts are ongoing to establish such facilities in Nigeria but there are challenges to establishing these services and making them sustainable [6-9]. There is currently no Open Heart Surgery (OHS) programme in Nigeria with sustained activity.

There has been some progress in the private sector in Nigeria, with the establishment of private cardiac catheterization services in Lagos (Reddington Multispecialty Hospital). Additionally, a private diagnostic facility has also been established in Lagos (First Cardiology Consultants). With the advent of these services, patients suspected of having Ischaemic Heart Disease (IHD) are being referred to First Cardiology Consultants (FCC) and where coronary angiography is indicated as part of the cardiac evaluation this is performed at the Cardiac Catheterization suite at the Reddington Multispecialty Hospital (RMH).

Without the support of Open Heart Surgery programmes in Nigeria difficult choices need to be made when patients that present with Ischaemic Heart Disease are found to have significant coronary artery disease. Where Percutaneous Coronary Intervention PCI is performed, this currently has to be done without surgical back-up.

The aim of this study was to describe the results of stand-alone PCI as currently practiced in Nigeria.

## Methods

The study population was all patients that underwent Cardiac catheterization at RMH as part of cardiac evaluation for IHD. Usual vascular access was via the right femoral artery. Coronary Ostia were cannulated and contrast injected to image and assess the coronary arteries to determine if intervention was required. Vessels that had tight ostial stenosis or were heavily calcified were deemed unsuitable for PCI and were referred abroad for Coronary Artery Bypass Grafting (CABG). Where the vessels were diffusely diseased the patients were continued on medical therapy. Major epicardial vessels or their branches with stenosis >70% or Left Main Stem Stenosis >50% were defined as significant stenoses which would require intervention [10]. These vessels underwent PCI with PTCA ± stenting. Stents used were either Drug Eluting Stents or Bare Metal Stents. Following PCI, the patients were monitored overnight in the high dependency unit to exclude any complications, prior to discharge home.

Following approval by the ethical committee of FCC data was extracted from a prospectively maintained database. The study period was from July 2009 till July 2012. The data extracted for analysis was that of Nigerian patients referred with a diagnosis of suspected IHD who went on to have cardiac catheterization and coronary angiography as part of their evaluation. Extracted data included patient demographics, euroscore, indication for cardiac catheterization, distribution and severity of coronary stenoses, type of PCI performed completeness of revascularization, result of PCI and complications seen.

Data analysis was performed with Microsoft Excel 2010 and results are expressed as numbers, mean ± standard deviation or percentages as appropriate.

## Results

152 Nigerians were referred with a diagnosis of IHD of which 80 were confirmed at coronary angiography to have Coronary Artery Disease (CAD). These 80 patients with confirmed CAD were the focus of this study. The sex distribution of patients with CAD was 53 males (66.2%) and 27 females (35.3%). The average age was 60.3 +/− 9.6 years. The average euroscore was 4.5 +/− 3.1.

The 80 patients with CAD had a total of 207 coronary stenoses, of which 171 (82.6%) were significant stenoses >70% (Table 1). These significant stenoses occurred in 77 patients (96.3%) who were therefore candidates for coronary revascularization. Distribution of the significant stenoses in these 77 patients showed one significant stenosis in 32 patients (41.5%), two significant stenoses in 11 patients (14.3%), three significant stenoses in 19 patients (24.7%), four significant stenoses in 13 patients (16.9%) and five significant stenoses in 2 patients (2.6%).

**Table 1:** Distribution of significant coronary stenoses and percutaneous coronary interventions

**Vessel**	**Stenosis**	**SS (%)**	**PTCA**	**BMS**	**DES**	**PCI total (% of SS)**
LMS	9	9 (100)	-	-	3	3 (33.3)
LAD Proximal	45	41 (91.1)	1	12	7	20 (48.8)
LAD Mid	24	22 (91.7)	2	2	8	12 (54.5)
LAD Distal	3	2 (66.7)	-	-	-	-
Diagonal	8	8 (100)	-	-	1	1 (12.5)
Cx Proximal	24	20 (83.3)	1	1	2	4 (20)
Cx Mid	13	9 (69.2)	-	6	-	6 (66.7)
Cx Distal	6	3 (50)	1	1	1	3 (100)
OM1	12	7 (58.7)	1	-	-	1 (14.3)
OM2	3	2 (66.7)	-	-	-	-
Intermediate	4	3 (75)	-	-	1	1 (33.3)
RCA Prox	27	21 (77.8)		3	2	8 (38.1)
RCA Mid	14	13 (92.8)	2	5	1	8 (61.5)
RCA Distal	7	5 (71.4)	1	1	1	3 (60)
PDA	4	3 (75)	1	1	-	2 (66.7)
PLB	4	3 (75)	-	-	-	-
**Total**	**207**	**171 (82.6)**	**13**	**32**	**27**	**72 (42.1)**

LAD: Left Anterior Descending Coronary Artery. Cx: Circumflex Coronary Artery. RCA: Right Coronary Artery. OM: Obtuse Marginal Coronary Artery. PDA: Posterior Descending Coronary Artery. PLB: Posterolateral branch of Right Coronary Artery. SS: Significant Stenosis. PTCA: Percutaneous Transluminal Coronary Angioplasty. BMS: Bare Metal Stent. DES: Drug Eluting Stent. PCI: Percutaneous Coronary Intervention.

PCI was performed in 48 patients (62.3%) but 29 patients (37.7%) did not have PCI. Of the 29 patients that did not have PCI, 28 (96.6%) had coronary anatomy unsuitable for PCI, while 1 patient (3.4%) had a pacemaker implanted for complete heart block. The indications for PCI are shown in Table 2. This shows that 39 of the 48 PCIs performed (81.2%) were for patients presenting urgently with myocardial infarction or unstable Angina.

**Table 2:** Indications for PCI and percentage of patients that underwent PCI

	**STEMI**	**NSTEMI/UA**	**Chronic stable angina**	**CCF**	**Preop workup**	**Total**
**Indication**	31	20	20	5	1	77
**PCI**	25	14	7	2	0	48
**%PCI**	80.6	70	35	40	0	62.3

STEMI: ST elevation myocardial Infarction. NSTEMI: Non-ST elevation myocardial infarction. UA: Unstable Angina. CCF: Congestive Cardiac Failure.

For the 48 patients that underwent PCI, 22 patients (45.8%) had one significant stenosis, 6 patients (12.5%) had two significant stenoses, 14 patients (29.2%) had three significant stenoses, 5 patients (10.4%) had four significant stenoses and 1 patient (2.1%) had five significant stenoses. Analysis of the completeness of revascularization (intervention to all significant stenoses) showed that complete revascularization was achieved in 100% of patients with single stenosis, 50% of patients with two stenoses, 21.4% of patients with three stenoses, 20% of patients with four stenoses and 0% of patients with five stenoses (Table 3). Overall 29 of the 48 patients (60.4%) had complete revascularization.

**Table 3:** Distribution of patients having PCI by the number of significant stenoses

	**One SS**	**Two SS**	**Three SS**	**Four SS**	**Five SS**	**Total**
**PCI to One Stenosis**	22	3	6	1	-	32
**PCI to Two Stenoses**	-	3	5	1	1	10
**PCI to Three Stenoses**	-	-	3	2	-	5
**PCI to Four Stenoses**	-	-	-	1	-	1
**PCI to Five Stenoses**	-	-	-	-	-	-
**Total PCI performed**	22	6	14	5	1	48
**% complete revascularization**	100	50	21.4	20	0	

PCI: Percutaneous Coronary Intervention. SS: Significant Stenosis.

PCI was performed by PTCA plus stenting in 41 patients (85.4%) and PTCA alone in 7 patients (14.6%). A total of 59 stents were used, with 32 (54.2%) being Bare Metal Stents and 27 (45.8%) being Drug Eluting Stents (Table 1).

Complications of PCI were bleeding that required blood transfusion in 1 patient (2.1%), minor femoral haematomas in 2 patients (4.2%), and a major adverse clinical event (MACE) in 1 patient (2.1%). The patient with MACE presented in shock from acute coronary syndrome and preoperative intra-aortic balloon pumping was instituted for LV assist. At cardiac catheterization a lesion in the RCA could not be crossed and the artery was unfortunately dissected. The patient was transferred urgently to a centre in Lagos for CABG which was performed with a reversed saphenous vein graft to the RCA but the patient died of progressive right ventricular failure [8].

## Discussion

In the early days of PCI there was a mortality rate of 1–2.5% and 1.9-5.8% of patients would proceed to emergency CABG [11-13]. Over the intervening years, as practice has improved, both the mortality rate and patients proceeding to emergency CABG is now <0.4% [14]. PCI is therefore now much safer and the indications for PCI extend to both patients with chronic stable angina as well as patients with myocardial infarction and acute coronary syndrome [14-16]. The growth in PCI has been in tandem with the growth in Open Heart Surgery programmes such that the choice of PCI or CABG can be made for the individual patient on the basis of presentation and the type of coronary lesions found. Referrals for elective as well as emergency CABG have subsequently dropped as PCI programmes have developed and are showing improving results [17,18]. The Achilles’ heel of PCI in the early days was a high restenosis rate [19] but careful patient selection and the development of Drug-Eluting stents has markedly reduced restenosis rates [20,21]. This has gradually made PCI the first choice for coronary revascularization for suitable coronary lesions [22].

Though the complications of PCI that would require emergency CABG have been reduced to a minimum in high volume centers, they do occur and it has been advised that PCI programmes should run alongside CABG programmes to prevent undue delay in the event of complications [23,24]. PCI and CABG programmes often co-exist in the same institution but this is not always the case, especially in rural areas where resources are more limited. This has therefore led to the debate as to whether stand-alone PCI programmes should be encouraged or whether the envelope is being pushed too far [25]. This debate continues and various guidelines have been released in different countries to guide the practice and obtain the best outcomes for patients. Most of the guidelines recommend that standalone PCI programmes should be within 5 minutes of a CABG programme and should be run by a high volume operator who is experienced in determining whether coronary stenoses are suitable for PCI or should be referred for CABG [23,25]. The 2005ACC/AHA/SCAI PCI guidelines recommended that primary PCI for STEMI should be performed by high volume operators experienced in both elective PCI and primary PCI for STEMI with ongoing activity levels of greater than 75 elective PCI a year and ideally annual PCI for STEMI of at least 11 a year [22].

Where do things stand in a developing country like Nigeria? The development of Open Heart Surgery and Cardiac Catheterization has been very slow in light of the peculiar challenges in Nigeria [6-9]. Open Heart Surgery has developed in fits and starts and there is currently no programme in Nigeria with regular sustained activity. Similarly, there has been no Cardiac Catheterization for several years till the advent of the laboratory at RMH in Lagos. In light of the absence of Open Heart Surgery support, should a Cardiac Catheterization facility be developed without the back-up of the possibility of emergency CABG? The findings from this study would suggest that there is certainly a place for stand-alone PCI in Nigeria. The first author was a high volume operator in practice in the US with an excess of 500 PCI cases annually including elective PCI and PCI for STEMI. Of the 77 patients with significant stenoses, 48 patients (62.3%) were selected as having lesions suitable for PCI. Over 80% of the PCI were performed for urgent cases with Myocardial Infarction and Unstable Angina. The complication rate was low with only one patient having MACE and there were no mortalities from PCI. The majority of patients undergoing PCI (85.4%) received stents of which Drug Eluting Stents was almost 50%.

Analysis of the results however underlines the urgency in pushing the concomitant development of Open Heart Surgery to make the option of CABG available so that patients with IHD and significant coronary stenoses can have the best outcomes. The choice of the best option for the patient can then be made on the basis of complexity of the lesion as obtains in the Western World. Out of the 77 patients found to have significant stenoses at coronary angiography, 28 patients (36.4%) had lesions too complex for PCI and had to be referred abroad for CABG. Although 48 patients underwent PCI, only 29 (60.4%) had complete revascularization. Of 171 stenoses identified as being significant, only 72 (42.1%) underwent PCI. All of the patients with single stenosis underwent PCI but the revascularization rate reduced as the number of significant stenoses increased. It is likely that the revascularization rate would have been considerably higher if an Open Heart Surgery Programme was available as those patients with more complex lesions would have also received surgical intervention. It has been suggested that PCI is an effective treatment for patients with multivessel disease without the need to dilate all diseased vessels with a reasonable expectation of satisfactory long-term clinical improvement [19] but this remains to be confirmed on follow up of this cohort of patients. The recently published results of the SYNTAX Trial [26] indicate that CABG results in reduced MACCE for coronary lesions with intermediate and high SYNTAX scores, whereas for lesions with low SYNTAX scores PCI is a good alternative as there is no difference in MACCE compared to CABG.

There are currently no other centres in West Africa offering PCI services. Volumes of PCI reported in this study were low as this reflects a new, developing service in Nigeria. Increased awareness of the existence of this service is already resulting in more referrals from physicians. In addition, the volumes were low due to careful case selection in the absence of back-up Open Heart Surgery services.

## Conclusion

A stand-alone PCI programme has been developed in Lagos, Nigeria. Both elective and urgent PCIs have been performed with no mortalities and a low complication rate. Increased volumes will however accrue and complete revascularization rates would be improved with the establishment of Open Heart Surgery programmes to provide CABG as back-up for PCI and alternate therapy for more complex lesions.

## Abbreviations

PCI: Percutaneous coronary intervention; PTCA: Percutaneous transluminal coronary angioplasty; CABG: Coronary artery bypass grafting; OHS: Open heart surgery; IHD: Ischaemic heart disease; CAD: Coronary artery disease; MACE: Major adverse cardiovascular events; LV: Left ventricle; RCA: Right coronary artery; ACC: American College of Cardiology; AHA: American heart association; SCAI: Society for cardiovascular angiography and interventions; STEMI: ST Elevation myocardial infarction; SYNTAX Trial: Synergy between PCI with Taxus and Cardiac Surgery Trial; MACCE: Major adverse cardiovascular and cerebral events; LAD: Left anterior descending coronary artery; Cx: Circumflex coronary artery; RCA: Right coronary artery; OM: Obtuse marginal coronary artery; PDA: Posterior descending coronary artery; PLB: Posterolateral branch of right coronary artery; SS: Significant stenosis; BMS: Bare metal stent; DES: Drug eluting stent; UA: Unstable angina.

## Competing interests

The authors declare that they have no competing interests.

## Authors' contributions

AJ performed all the Percutaneous Coronary Interventions, reviewed the first draft, assisted with data extraction and made intellectual contributions. BF conceived of the study, extracted the patient data, did the data analysis, wrote the first draft, and reviewed the second draft. IA assisted with the extraction of patient data, assisted with the data analysis and reviewed the second draft. YO reviewed the final draft and made intellectual contributions. All the authors reviewed and approved the final draft for submission.

## Pre-publication history

The pre-publication history for this paper can be accessed here:

http://www.biomedcentral.com/1471-2261/14/8/prepub
